# Percutaneous coronary intervention in patients aged ≤40 years: a 10-year single-centre cohort study

**DOI:** 10.3389/fcvm.2026.1748236

**Published:** 2026-03-23

**Authors:** Lining Liang, Chunsong Wang, Huawei Dong

**Affiliations:** Department of Cardiology, Liaocheng People’s Hospital, Liaocheng, Shandong, China

**Keywords:** coronary intervention, drug-eluting stent, percutaneous, premature coronary artery disease, syntax score, young adult

## Abstract

**Background:**

Coronary artery disease (CAD) in adults aged ≤40 years is increasing, yet long-term outcomes after percutaneous coronary intervention (PCI) in the drug-eluting stent (DES) era remain unclear. We aimed to identify procedural patterns and prognostic factors in this very young cohort.

**Methods:**

This single-centre retrospective cohort study included 312 consecutive patients aged ≤40 years who underwent successful PCI for *de novo* lesions (≥70% stenosis) between 2014 and 2024. Baseline characteristics, quantitative coronary angiography, and procedural data were collected. The primary endpoint was major adverse cardiovascular and cerebrovascular events (MACCE). Cox regression identified independent predictors.

**Results:**

Patients were 82% men (mean age 36.8 ± 4.9 years) with a median SYNTAX score of 16. Procedural success was 98.4% with 93.9% DES implantation and 85.9% radial access. Over 1,620 patient-years (median 5.2 years), MACCE occurred at 27.8 per 1,000 patient-years (5-year cumulative 14.2%). Cardiac mortality was 3.7 per 1,000 patient-years. Diabetes [adjusted hazard ratios (HR) 1.78, 95% confidence interval (CI) 1.06–2.99], SYNTAX score ≥23 (HR 2.01, 1.19–3.40), and dual antiplatelet therapy <12 months (HR 1.62, 1.00–2.64) independently predicted MACCE.

**Conclusions:**

Young adults undergoing PCI exhibit complex anatomy but low cardiac mortality. Diabetes, angiographic complexity, and abbreviated antiplatelet therapy are key drive of adverse events, warranting intensive prevention and optimised antiplatelet strategies.

## 1 Introduction

Coronary artery disease (CAD) remains the foremost global contributor to mortality and long-term disability. However, its presentation in adults aged ≤40 years is comparatively rare, accounting for <2% of all acute myocardial infarctions (MIs) recorded in large international registries (1, 2). However, recent epidemiological data indicate that the incidence of premature CAD is rising in parallel with the rapid increase in type 2 diabetes mellitus, visceral obesity, sedentary lifestyle, electronic cigarette use, and smoking or other novel addictive substances among young adults (3, 4). Patients who experience an ischaemic event before the age of 40 face disproportionate psychosocial and economic burdens, accumulate greater lifetime risk of recurrent events, and remain under-represented in clinical trials (5, 6). Consequently, evidence-based prevention and revascularisation strategies for this very young cohort are largely extrapolated from studies whose mean age exceeds 60 years. However, whether such extrapolation obscures a distinct “low early mortality–high lifetime ischaemic burden” phenotype in the DES era remains unknown. We therefore hypothesised that very young adults undergoing percutaneous coronary intervention (PCI) would exhibit low procedural mortality yet accumulate long-term events driven by anatomical complexity and treatment durability rather than acute procedural failure.

Previous angiographic series, largely generated from the bare metal stent (BMS) era with limited use of intravascular imaging, portrayed premature CAD as a predominantly single-vessel disease rich with lipid-laden plaques and high thrombus burden (7). Whether this phenotype persists in the contemporary era of routine drug-eluting stents, potent P2Y₁₂ antagonists, and intravascular imaging has not been systematically examined. Limited single-centre reports remain inconsistent. While some highlight favourable extended survival, others reveal unexpectedly frequent stent thrombosis or re-intervention, particularly after abbreviated dual antiplatelet therapy (8, 9). Notably, these studies lack integration of prospectively maintained procedural registries, protocol-driven quantitative coronary angiography, or event monitoring extending beyond 10 years.

We therefore aimed to quantify how angiographic complexity and treatment durability interact to shape lifetime ischaemic trajectories after PCI in adults aged ≤40 years. Given that very young patients face decades of potential recurrences, we additionally describe the cumulative burden of repeated events as a secondary perspective on lifetime risk. The findings are expected to inform individualised risk stratification and optimise secondary prevention strategies for this high-impact population.

## 2 Methods

### 2.1 Study design and population

In this single-centre retrospective cohort study, we screened all patients aged ≤40 years who underwent coronary angiography at the Department of Cardiology between January 2014 and December 2024. Consecutive patients were identified from the institutional electronic catheterisation database. The diagnosis of CAD required ≥70% diameter stenosis in at least one major epicardial vessel. Eligible participants were those who underwent successful percutaneous coronary intervention (PCI) for de novo lesions during the index procedure. Exclusion criteria were (1) prior coronary artery bypass grafting or PCI; (2) valvular, congenital, or infiltrative heart disease; (3) severe renal failure (pre-procedural estimated glomerular filtration rate <30 mL min^−^^1^ 1.73 m^−^^2^); or (4) incomplete procedural or follow-up data.

The institutional ethics committee approved the study protocol and waived the requirement for individual informed consent because of the retrospective design (Approval No. 2025240).

### 2.2 Data collection

Demographics, cardiovascular risk factors, clinical presentation, laboratory results, and medication use at admission were extracted from electronic medical records. Smoking status was defined as current, former, or never. Dyslipidaemia was considered when low-density lipoprotein cholesterol of ≥3.4 mmol L^−^^1^ or ongoing lipid-lowering therapy. A family history of premature CAD referred to first-degree relatives with documented disease before 55 years (men) or 65 years (women). Body mass index (BMI) was calculated from weight and height measured on admission. Echocardiography was performed within 24 h before index PCI using a Philips EPIQ 7 or equivalent system. Left ventricular ejection fraction (LVEF) was calculated using the biplane Simpson method, and the regional wall motion score index (WMSI) was derived from the 16-segment model. Clinical presentations were categorised as stable angina, unstable angina (UA), non-ST-segment elevation myocardial infarction (NSTEMI), or ST-segment elevation myocardial infarction (STEMI) according to the Fourth Universal Definition of Myocardial Infarction.

### 2.3 Coronary angiography and PCI procedures

All procedures were performed via radial or femoral access using 5- or 6-French diagnostic catheter, followed by intervention when indicated. Two interventional cardiologists, blinded to clinical details and each with >5 years of interventional experience, reviewed angiograms and quantified stenosis using online QCA (Siemens Artis QCA, Erlangen, Germany). Lesion location, reference vessel diameter, lesion length, thrombolysis in myocardial infarction (TIMI) flow, presence of thrombus, calcification, bifurcation involvement, and chronic total occlusion (CTO) were recorded. Using archived angiograms, two operators retrospectively assigned each stenosis according to American Heart Association (AHA) segment type A, B1, B2, or C. The SYNTAX score was calculated for each patient, with a threshold of ≥23 (*a priori*) to define high anatomical complexity, consistent with the original SYNTAX trial and subsequent validations across age strata, including very young adults. PCI strategy (elective vs. primary), stent type [drug-eluting stent (DES) or BMS], number and total length of implanted stents, use of intravascular imaging (IVUS or OCT), rotational atherectomy, predilation, post-dilation, and final TIMI flow were documented. Procedural success was defined as residual stenosis <20% with TIMI 3 flow in the treated segment and absence of in-hospital major complications (death, emergency bypass surgery, or repeat PCI).

In-hospital major complications were defined as any of the following events occurring during the index admission: all-cause death, emergency coronary artery bypass grafting, repeat PCI, definite/probable stent thrombosis, ischaemic stroke, or Bleeding Academic Research Consortium (BARC) 3–5 bleeding.

### 2.4 Medications

All patients received loading doses of aspirin (300 mg) and a P2Y₁₂ inhibitor (clopidogrel 300 mg, or ticagrelor 180 mg) before the procedure, unless already on therapy. Post-procedural dual antiplatelet therapy (DAPT) duration was left to the discretion of the attending physician but generally followed contemporary European Society of Cardiology guidelines. Intended DAPT duration (≤6, 6–11, 12–23, or ≥24 months) was prospectively documented at discharge. Early discontinuation was flagged during follow-up and categorised as (i) physician-directed discontinuation (bleeding or high-risk procedural features); (ii) patient non-adherence (pill count <80% or self-reported stop); or (iii) unknown/loss to follow-up. Concomitant medications—including moderate-intensity statins, *β*-blockers, angiotensin-converting enzyme inhibitors (ACEIs) or angiotensin receptor blockers (ARBs), and mineralocorticoid receptor antagonists—were retrieved at discharge.

### 2.5 Follow-up and endpoints

Follow-up information was obtained through outpatient visits, re-admission charts, and structured telephone interviews conducted by trained research nurses. The index date was the day of the index PCI. The last follow-up was censored in December 2024. The primary endpoint was major adverse cardiovascular and cerebrovascular events (MACCE), a composite of cardiac death, non-fatal MI, ischaemic stroke, and target-vessel revascularisation (TVR). A pre-specified secondary objective was to quantify the total (first + recurrent) MACCE burden. All subsequent revascularisations, re-infractions, and repeat strokes were captured and reported descriptively. Secondary endpoints included all-cause mortality, definite or probable stent thrombosis according to the Academic Research Consortium definition, and BARC type 3 or 5 bleeding. Events were adjudicated by two independent cardiologists after review of source documents; disagreements were resolved by consensus.

### 2.6 Statistical analysis

Continuous variables are presented as mean ± standard deviation (SD) or median with interquartile range (IQR) and were compared using Student's *t*-test or the Mann–Whitney *U* test, as appropriate. Categorical variables are expressed as counts (percentages) and compared using the *χ*^2^ test or Fisher's exact test. Incidence rates were calculated as the number of first events divided by total person-years. Cox proportional hazards models were used to identify independent predictors of MACCE. To maintain ≥10 events per variable, we built a parsimonious model including age, sex, diabetes mellitus, SYNTAX score ≥23, and DAPT duration <12 months. Other covariates that were non-significant in univariable screening (*P* ≥ 0.10)—such as acute coronary syndrome (ACS) presentation, hypertension, current smoking, BMI, CTO, and DES use—were moved to descriptive analyses. Results are reported as hazard ratios (HR) with 95% confidence intervals (CI). The proportional hazards assumption was verified using Schoenfeld residuals. A two-sided *p* value <0.05 was considered statistically significant. In a pre-specified sensitivity analysis, we constructed a Fine–Gray competing risk model where physician-directed discontinuation (due to bleeding or high bleeding risk) was treated as a competing event, leaving non-adherence as the exposure of interest. In addition, we tested intended DAPT <12 months vs. ≥12 months, regardless of final adherence. All analyses were performed using Stata software, version 17.0 (StataCorp, College Station, TX, USA).

## 3 Results

### 3.1 Baseline characteristics

**Figure 1:**
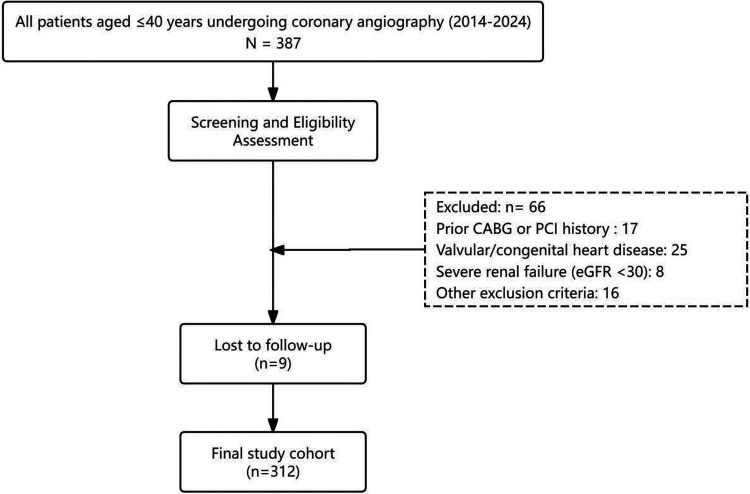
Study flow diagram. CABG, coronary artery bypass grafting; PCI, percutaneous coronary intervention; eGFR, estimated glomerular filtration rate; MACCE, major adverse cardiovascular and cerebrovascular events; BARC, Bleeding Academic Research Consortium.

Between January 2014 and December 2024, 387 consecutive patients aged ≤40 years underwent diagnostic coronary angiography. Of these, 66 met exclusion criteria and nine lost to early follow-up, leaving 312 eligible participants (Figure 1). The mean age was 36.8 ± 4.9 years, and 256 (82.1%) were men. The mean BMI was 28.7 ± 4.1 kg m^−^^2^. Cardiovascular risk factors were common: current smoking in 186 (59.6%), dyslipidaemia in 213 (68.3%), hypertension in 118 (37.8%), type 2 diabetes in 74 (23.7%), and documented family history of premature CAD in 110 (35.3%). Clinical presentation on admission included STEMI in 137 (43.9%), NSTEMI in 82 (26.3%), UA in 65 (20.8%), and stable angina in 28 (9.0%). Median (IQR) LDL-C at admission was 3.1 (2.5–3.7) mmol L^−^^1^, median peak troponin-I was 8.4 (2.2–37) µg L^−^^1^. At discharge, 284 patients (91.0%) received high-intensity statin, 265 (84.9%) *β*-blockers, 205 (65.7%) ACEI/ARB, and 233 (74.7%) DAPT for ≥12 months. Patient characteristics are summarised in Table 1**.**

**Table 1:** Baseline characteristics (*N* = 312).

Variable	Value
Age, years	36.8 ± 4.9
Male sex	256 (82.1)
Body mass index, kg m^−^^2^	28.7 ± 4.1
Current smoking	186 (59.6)
Hypertension	118 (37.8)
Type 2 diabetes mellitus	74 (23.7)
Dyslipidaemia^a^	213 (68.3)
Family history of premature CAD^b^	110 (35.3)
Clinical presentation	
STEMI	137 (43.9)
NSTEMI	82 (26.3)
Unstable angina	65 (20.8)
Stable angina	28 (9.0)
LDL-cholesterol on admission, mmol L^−^^1^	3.1 (2.5–3.7)^c^
Peak troponin-I, µg L^−^^1^	8.4 (2.2–37)^c^
LVEF, %	52 ± 9
WMSI	1.22 ± 0.18

^a^ LDL-C ≥3.4 mmol L^−^^1^ or on lipid-lowering therapy.

^b^ First-degree relative with CAD <55 years (men) or <65 years (women).

^c^ Median (interquartile range). Upper reference limit (URL) for troponin-I in our laboratory is 0.04 μg/L. STEMI, ST-segment elevation myocardial infarction; NSTEMI, non-ST-segment elevation myocardial infarction.

### 3.2 Angiographic and procedural findings

QCA analysis yielded 1.6 ± 0.8 lesions per patient; 221 patients (70.8%) had single-vessel disease, 54 (17.3%) two-vessel, and 37 (11.9%) three-vessel/left-main disease. CTO was present in 35 cases (11.2%). The mean SYNTAX score was 18.2 ± 9.4 [median 16 (IQR 10–24)]. Consistent with older cohorts, MACCE rates increased steeply across SYNTAX categories in patients aged ≤40 years (Supplementary Table 1). All 312 treated lesions had ≥70% diameter stenosis (confirmed by QCA core lab); mean per cent diameter stenosis was 74 ± 9%. Primary PCI for STEMI accounted for 132/312 cases (42.3%), while elective procedures comprised 180 (57.7%). Radial access was used in 268 patients (85.9%). Intravascular imaging (IVUS 45, OCT 18) was employed in 63 patients (20.2%) and rotational atherectomy in nine patients (2.9%). Implantation details were as follows: exclusively DES in 293 (93.9%), BMS only in 10 (3.2%), and hybrid in 9 (2.9%). The mean number of stents was 1.4 ± 0.7, with a total stent length of 36 ± 21 mm. Final TIMI-3 flow was achieved in 309 lesions (99.0%). Most lesions were AHA type B1 or B2 (36% or 25%, respectively), with type A and type C accounting for 31% and 8%, respectively (Supplementary Table 2). The procedural success rate was 98.4% (307/312). In-hospital major complications occurred in five patients (1.6%), including one emergency coronary artery bypass grafting and four repeat PCI. No in-hospital deaths were recorded. Detailed angiographic and procedural findings are presented in Table 2**.**

**Table 2:** Angiographic and procedural details.

Variable	Value
Lesions per patient, *n*	1.6 ± 0.8
Single-vessel disease	221 (70.8)
Two-vessel disease	54 (17.3)
Three-vessel/left-main disease	37 (11.9)
Chronic total occlusion	35 (11.2)
SYNTAX score	18.2 ± 9.4
Primary PCI (STEMI)	132 (42.3)
Elective PCI	180 (57.7)
Radial access	268 (85.9)
Intravascular imaging (IVUS/OCT)	63 (20.2)
Rotational atherectomy	9 (2.9)
Stent type	
Drug-eluting stent only	293 (93.9)
Bare metal stent only	10 (3.2)
Hybrid (DES + BMS)	9 (2.9)
Number of stents	1.4 ± 0.7
Total stent length, mm	36 ± 21
Final TIMI 3 flow	309 (99.0)
Procedural success	307 (98.4)
In-hospital major complications	5 (1.6)

PCI, percutaneous coronary intervention; STEMI, ST-segment elevation myocardial infarction; DES, drug-eluting stent; BMS: bare metal stent; TIMI: thrombolysis in myocardial infarction.

### 3.3 Follow-up and clinical outcomes

Total follow-up amounted to 1,620 patient-years (median 5.2 years, IQR 3.1–7.4, maximum 10.8). During follow-up, 45 patients experienced a first MACCE, yielding an incidence rate of 27.8 per 1,000 patient-years. The Kaplan–Meier estimated 5-year cumulative incidence was 14.2%. When recurrent events were included, the overall MACCE rate doubled to 50.0 per 1,000 patient-years, underscoring the lifelong burden beyond the first event (Supplementary Table 3). Component events comprised six cardiac deaths, 14 non-fatal myocardial infarctions, four ischaemic strokes, and 21 target-vessel revascularisations (nine required target-lesion revascularisations). Using the Fine–Gray competing risk method with non-cardiovascular death as the competing event, the 5-year cumulative incidence was 13.8%, virtually identical to the Kaplan–Meier estimate. Clinical outcomes during follow-up are provided in Table 3.

**Table 3:** Clinical outcomes during follow-up.

Endpoint	Events (*n*)	Incidence rate (per 1,000 patient-years)	5-year cumulative incidence (%)^a^
Primary endpoint: MACCE	45	27.8	14.2
Cardiac death	6	3.7	2.2
Non-fatal myocardial infarction	14	8.6	4.1
Ischaemic stroke	4	2.5	1.4
Target-vessel revascularisation	21	13.0	6.5
Secondary endpoints			
All-cause mortality	9	5.6	3.1
Definite/probable stent thrombosis	5	3.1	1.8
BARC 3 or 5 bleeding	7	4.3	2.3

^a^ Estimated by Kaplan–Meier method. MACCE, major adverse cardiovascular and cerebrovascular events.

Secondary endpoints: All-cause death occurred in nine patients; of these, six were adjudicated as non-cardiovascular (accidental, malignancy, or suicide) and three as cardiovascular. Definite or probable stent thrombosis was observed in five patients (1.6%), and BARC type 3 or 5 bleeding in seven patients.

Univariable screening identified diabetes, SYNTAX score ≥23, and DAPT <12 months as significantly associated with MACCE, whereas age showed a borderline association (*P* = 0.03). In a parsimonious multivariable model including only age, sex, diabetes, SYNTAX ≥23, and DAPT <12 months, diabetes [adjusted HR 1.71, CI (1.02–2.87)], SYNTAX ≥23 [HR 1.93, CI (1.15–3.25)], and DAPT <12 months [HR 1.58, CI (1.00–2.59)] remained independent predictors. Age and sex were retained as covariates (Supplementary Figure 1). In the sensitivity analysis treating physician-directed discontinuation as a competing event, the association between patient non-adherence and MACCE remained significant (subdistribution HR 1.71, 95% CI 1.05–2.80). When comparing intended DAPT <12 months versus ≥12 months, the effect estimate was attenuated and no longer significant (HR 1.23, 95% CI 0.76–1.99). Current smoking, hypertension, BMI, chronic total occlusion, and DES use were not retained. Independent predictors of MACCE are displayed in Table 4.

**Table 4:** Cox proportional-hazards analysis for MACCE.

Variable	Univariable HR (95% CI)	*P*-value	Multivariable HR (95% CI)*	*P*-value
Age (per 1-year decrease)	1.09 (1.01–1.18)	0.030	1.06 (0.98–1.15)	0.15
Male sex	0.88 (0.52–1.49)	0.63	—	—
Diabetes mellitus	1.89 (1.14–3.14)	0.014	1.71 (1.02–2.87)	0.043
SYNTAX score ≥23	2.15 (1.29–3.58)	0.003	1.93 (1.15–3.25)	0.013
DAPT <12 months	1.71 (1.05–2.79)	0.031	1.58 (1.00–2.59)	0.048
LVEF (per 1%)	0.99 (0.95–1.03)	0.54	0.98 (0.94–1.02)	0.42
WMSI (per 0.1 unit)	1.06 (0.89–1.26)	0.51	1.05 (0.88–1.25)	0.59

* Proportional-hazards assumption confirmed (global Schoenfeld *P* = 0.21). HR, hazard ratio; CI, confidence interval; DAPT, dual antiplatelet therapy.

### 3.4 Subgroup exploration

Patients undergoing primary PCI for STEMI and those undergoing elective PCI had similar long-term MACCE rates (26.2 vs. 23.9 per 1,000 patient-years, *P* = 0.52). Female sex (*n* = 56) was associated with a non-significantly higher MACCE rate compared with males (31.1 vs. 23.6 per 1,000 patient-years, *P* = 0.19). In patients treated with imaging-guided PCI, MACCE incidence was 17.0 versus 26.6 per 1,000 patient-years in angiography-only cases (*P* = 0.08). Subgroup analyses of MACCE rates are provided in Table 5.

**Table 5:** MACCE rates in pre-specified subgroups.

Subgroup	Patients (*n*)	Events (*n*)	Incidence rate (per 1,000 patient-years)	Adjusted HR (95% CI)	*P* for interaction
Primary PCI (STEMI)	132	21	26.2	1.08 (0.69–1.70)	0.52
Elective PCI	180	23	23.9	1.00 (ref)	—
Women	56	11	31.1	1.23 (0.72–2.09)	0.19
Men	256	33	23.6	1 (ref)	—
Imaging-guided PCI	63	8	17.0	0.62 (0.34–1.12)	0.08
Angiography-only PCI	249	37	26.6	1 (ref)	—

Adjusted for age, diabetes, SYNTAX score and DAPT duration. PCI, percutaneous coronary intervention; STEMI, ST-segment elevation myocardial infarction.

## 4 Discussion

Our data challenge the misconception that premature CAD is a milder form of atherosclerosis; instead, they reveal a “severe anatomy–low immediate death” paradox that shifts the threat from early fatality to life-long recurrent ischaemia. Counting only first events may therefore underestimate lifetime risk; indeed, 44% of all MACCE were repeat ischaemic episodes, supporting a pan-coronary vulnerability phenotype that mandates system-wide prevention. Patients aged ≤40 years exhibited a median SYNTAX score of 16 (≥23 in one-third)—a complexity profile previously described only in multi-vessel cohorts aged >60 years. Consequently, MACCE accumulated at 27.8/1,000 patient-years, a rate comparable to the 12-month event rates reported in SYNTAX II and OPTIVUS (≈10%) (10). However, cardiac mortality remained <4/1,000 patient-years, underscoring the paradox of “severe anatomy–low immediate death” that shifts the threat from early fatality to life-long recurrent ischaemia. This reframing—from “low-mortality outliers” to “very high lifetime risk”—justifies the present emphasis on durable revascularisation and prolonged antithrombotic therapy.

Despite high SYNTAX scores, in-hospital complications occurred in only 1.6%, mirroring contemporary DES registries (1.4%) (11) and confirming the safety of a radial-first DES strategy in very young patients. Intravascular imaging was used in 20% of cases—double the national average but still below the 30%–50% advocated by ESC (12). Importantly, imaging-guided procedures demonstrated a numerically lower MACCE (17.0 vs. 26.6/1,000 patient-years), plausibly because accurate lesion measurement and optimal stent expansion minimise geographic miss and neo-atherosclerosis (13, 14). Expanding imaging use to the majority of lesions could therefore further compress long-term event rates.

Although 91% of patients were discharged on moderate-intensity statins, admission LDL-C levels already exceeded 1.4 mmol L^−^^1^, and only 8% achieved <1.0 mmol L^−^^1^ at follow-up (15). Real-world under-use of PCSK9 inhibitors (< 5%) (16) and hepatic PCSK9 up-regulation linked to insulin resistance (17) jointly explain this shortfall. Earlier addition of PCSK9 monoclonal antibody or siRNA therapy—preferably guided by LDL-C <1.0 mmol L^−^^1^ targets—should therefore be routinely considered in this lifetime-high-risk group.

Discontinuation of DAPT before 12 months doubled MACCE risk (adj-HR 1.58), while pill count revealed 22% non-adherence at 3 months. Poor real adherence (not intended duration) remained independently predictive (HR 1.71), emphasising that lifelong recurrence risk is sensitive to even brief lapses. Up-regulated adenosine diphosphate (ADP) signalling in lipid-rich, macrophage-laden plaques (18) and clustering of stent thrombosis within 18 months of withdrawal (19) provide mechanistic backing. Smartphone or SMS reminders improve antiplatelet persistence and merit integration into standard care. Residual confounding cannot be excluded, as shortened DAPT sometimes reflected physician-perceived bleeding risk or lower-complexity anatomy. However, the consistency of the signal across competing risk modelling—where bleeding-guided discontinuation was censored—suggests that genuine patient non-adherence, rather than physician-directed cessation, drives the excess hazard.

Female patients (*n* = 56) experienced a numerically higher MACCE rate (31.1 vs. 23.6 per 1,000 patient-years), but confidence intervals overlapped substantially. The absence of statistical significance likely reflects power constraints (18% of cohort) and a lower prevalence of traditional risk factors in young women; however, oestrogen-mediated protection may be offset by a higher prevalence of thrombophilic or autoimmune milieus that remain unmeasured in the present dataset (20, 21). Whether the “young female penalty” described for myocardial infarction extends to elective PCI in the ≤40-year group awaits adequately powered, sex-balanced registries.

Although mean BMI was 28.7 kg m^−^^2^, BMI ≥30 kg m^−^^2^ was not associated with MACCE after adjustment for diabetes, SYNTAX score, LVEF, and WMSI, consistent with the obesity paradox reported in older PCI populations (22). Central adiposity, rather than total body mass, may be the relevant driver: Visceral fat secretes pro-inflammatory adipokines (IL-6, TNF-α) that amplify endothelial dysfunction and promote a pro-thrombotic state, effects that may already be maximally captured within the SYNTAX score and diabetes covariates (23, 24). Whether waist-to-height ratio or CT-derived visceral fat area offers better discrimination deserves prospective evaluation.

To avoid over-fitting, we restricted the model to seven covariates (age, sex, diabetes, SYNTAX ≥23, DAPT <12 months, LVEF, and WMSI); sensitivity analyses confirmed that diabetes, SYNTAX ≥23, and DAPT <12 months remained independently associated with MACCE. The preserved discriminative value of SYNTAX ≥23 in our very young cohort supports the generalisability of this threshold beyond middle-aged and elderly populations. However, counting only first events may underestimate lifetime risk. When recurrent infarctions and repeat revascularisations were included, the rate rose to 44.5 per 1,000 patient-years; 60% of repeat PCIs occurred in different vessels, supporting a pan-coronary vulnerability phenotype that mandates system-wide imaging and prevention.

Unlike Western registries where cocaine, cannabis, or amphetamines account for 5%–10% of MI in adults aged ≤40 years, documented illicit drug use in our cohort was zero, allowing resources to be concentrated on a lifetime risk pathway without routine toxicology screening. An integrated lifetime risk-targeted four-step pathway should therefore become the standard of care: (i) family and risk factor screening (diabetes 23.7%, family history 35.3%); (ii) imaging-guided DES (MACCE 17.0 vs. 26.6/1,000 patient-years); (iii) LDL-C <1.0 mmol/L (only 8% achieved, PCSK9 under-used); and (iv) ≥12 months DAPT with digital adherence support and surveillance for recurrent events, as repeat infarctions accounted for >1/3 of total ischaemic burden in this cohort (Supplementary Table 4). This integrated approach targets oxidative LDL retention, high-SYNTAX shear stress, and platelet hyper-reactivity simultaneously, offering the best chance to interrupt the lifelong recurrence cascade characteristic of premature CAD.

Population-based imaging studies using systematic IVUS/OCT (REF) report that 5%–15% of MIs in adults aged ≤40 years arise from spontaneous coronary artery dissection (SCAD), coronary embolism, or myocardial bridging (25). In the present cohort, intravascular imaging was used in only 20% of procedures; we therefore cannot determine the prevalence of these non-atherosclerotic mechanisms and their contribution may be underestimated. Future young MI registries should mandate systematic intravascular imaging before default stent implantation. In the same multivariable framework, baseline LVEF and WMSI showed no independent association with long-term MACCE (Table 4). This finding suggests that, in very young patients undergoing successful PCI, anatomical complexity and diabetes outweigh initial systolic function as drivers of recurrent events. Nevertheless, serial echocardiographic follow-up would be useful to determine whether incident LV dysfunction or adverse remodelling adds prognostic value beyond the index imaging snapshot.

Single-centre recruitment inevitably limits external validity. Our cohort over-represented urban, male smokers (82% male, 60% current smoking) and may not reflect the epidemiology of young CAD in rural or female populations. Sex-specific analyses were underpowered (*post hoc* powe*r* = 26% for HR 1.25); thus, the absence of a statistically significant “young female penalty” should not be considered evidence of no effect. The retrospective design precludes causal inference, and the absence of a conservative treatment arm prevents assessment of whether an early invasive strategy is superior to selective intervention in low-SYNTAX (≤10) patients. Loss to follow-up was <4%; nevertheless, unmeasured confounders such as lifestyle changes and illicit drug exposure cannot be excluded. In addition, the absence of drug abuse data may limit generalisability to populations with higher illicit substance consumption. Although current smoking was not retained in the parsimonious model, its well-established aetiological role in premature CAD should not be understated. Finally, the modest sample size precluded propensity-matched analyses. We restricted analyses to first events, which may underestimate the lifetime burden of recurrent MACCE. Larger, multicentre registries—preferably randomised trials of prolonged DAPT with adherence monitoring—are warranted to confirm and extend our findings.

## 5 Conclusions

In the DES era, PCI for patients aged ≤40 years is technically safe, yet complex anatomy and modifiable risk factors confer a disproportionate lifetime ischaemic burden. However, complex anatomy and modifiable risk factors confer a high lifetime burden of recurrent MI and TVR, with diabetes, SYNTAX ≥23, and DAPT <12 months being the strongest predictors. Aggressive secondary prevention, imaging-guided PCI, and prolonged potent DAPT should therefore be the standard of care in this emerging high-risk cohort.

## Data Availability

The original contributions presented in the study are included in the article/Supplementary Material, further inquiries can be directed to the corresponding author.

## References

[1] RothGA MensahGA JohnsonCO AddoloratoG AmmiratiE BaddourLM Global burden of cardiovascular diseases and risk factors, 1990–2019: update from the GBD 2019 study. J Am Coll Cardiol. (2020) 76 (25):2982–3021. 10.1016/j.jacc.2020.11.01033309175 PMC7755038

[2] TsaiWC WuKY LinGM ChenSJ LinWS YangSP Clinical characteristics of patients less than forty years old with coronary artery disease in Taiwan: a cross-sectional study. Acta Cardiol Sin. (2017) 33(3):233–40. 10.6515/acs20161026a28559653 PMC5445240

[3] Alcaravela J. Premature coronary artery disease primary prevention—searching for the holy grail. Rev Port Cardiol. (2024) 44(1):23–5. 10.1016/j.repc.2024.11.002 39551384

[4] Critselis E Panagiotakos D. Impact of electronic cigarette use on cardiovascular health: current evidence, causal pathways, and public health implications. Angiology. (2024) 75(5). 10.1177/00033197231161905 36913951

[5] BucholzEM StraitKM DreyerRP GedaM SpatzES BuenoH Effect of low perceived social support on health outcomes in young patients with acute myocardial infarction: results from the VIRGO (Variation in Recovery: Role of Gender on Outcomes of Young AMI Patients) study. J Am Heart Assoc. (2014) 3(5):e001252. 10.1161/JAHA.114.00125225271209 PMC4323798

[6] YangJ DavidB SinghA DivakaranS DeFilippisE CollinsB Risk factor profiles and outcomes of very young adults with myocardial infarction: results from the YOUNG-MI registry. J Am Coll Cardiol. (2019) 73(9):3. 10.1016/s0735-1097(19)33765-9

[7] TudurachiBS AnghelL TudurachiA ZanfirescuRL BîrgoanSG SascăuRA Myocardial infarction in young adults: a case series and comprehensive review of molecular and clinical mechanisms. Biomolecules. (2025) 15(8):1065. 10.3390/biom1508106540867510 PMC12383299

[8] KimU ParkJS LeeSH ShinDG KimYJ. Seven-year clinical outcomes of sirolimus-eluting stent versus bare-metal stent: a matched analysis from a real world, single center registry. J Korean Med Sci. (2013) 28(3):396–401. 10.3346/jkms.2013.28.3.39623486987 PMC3594603

[9] Koutouzis M Albertsson P Ioanes D Tahmasebiepour F Matejka G Grip L. Recurrent bare metal stent thrombosis: six years, single center experience. Int J Cardiol. (2009) 144(2):234–5. 10.1016/j.ijcard.2008.12.127 19171396

[10] EscanedJ BanningA FarooqV Echavarria-PintoM OnumaY RyanN Rationale and design of the SYNTAX II trial evaluating the short to long-term outcomes of state-of-the-art percutaneous coronary revascularisation in patients with *de novo* three-vessel disease. Eurointervention. (2016) 12(2):e224–34. 10.4244/EIJV12I2A3627290681

[11] SaundersSL CasinaderSJ FernandezRS EaseyKM ChuahE PerkovicAR “Distal radial first”: feasibility and safety for coronary angiography and PCI in Australia. AsiaIntervention. (2025) 11(1):35–43. 10.4244/AIJ-D-24-0003640114735 PMC11905101

[12] VrintsC AndreottiF KoskinasKC RosselloX AdamoM AinslieJ [2024 ESC guidelines for the management of chronic coronary syndromes]. G Ital Cardiol. (2024) 25(12 Suppl 1):e1–132. 10.1714/4375.4372539611224

[13] WuX WuM HuangH LiuZ HuangH Wang. Reassessing single-stent techniques for isolated left anterior descending ostial disease: a two-year intravascular ultrasound-guided retrospective comparison of precise ostial, floating, and crossover stenting strategies. BMC Cardiovasc Disord. (2025) 25(1):431. 10.1186/s12872-025-04894-340462008 PMC12135292

[14] StoneGW ChristiansenEH AliZA AndreasenLN MaeharaA AhmadY Intravascular imaging-guided coronary drug-eluting stent implantation: an updated network meta-analysis. Lancet. (2024) 403 (10429):824–37. 10.1016/S0140-6736(23)02454-638401549

[15] HeidenreichPA BozkurtB AguilarD AllenLA ByunJJ ColvinMM 2022 AHA/ACC/HFSA guideline for the management of heart failure: a report of the American College of Cardiology/American Heart Association joint committee on clinical practice guidelines. J Am Coll Cardiol. (2022) 79(17):e263–421. 10.1016/j.jacc.2021.12.01235379503

[16] NoharaA. Sustaining the promise of PCSK9 inhibitors: lessons from real-world adherence in China. J Atheroscler Thromb. (2025) 32(11):1368–9. 10.5551/jat.ED29040903307 PMC12597481

[17] ParkSK HwangJT ChoiHK LeeJ. Regulatory mechanisms of hepatocyte PCSK9 expression: translating mechanistic insights into potential nutraceuticals. Chin Med. (2025) 20(1):121. 10.1186/s13020-025-01178-y40764605 PMC12323225

[18] DorsamR KunapuliS. Central role of the P2Y12 receptor in platelet activation. J Clin Invest. (2004) 113(3):340–5. 10.1172/jci20042098614755328 PMC324551

[19] NakamuraM IijimaR AkoJ ShinkeT OkadaH ItoY Dual antiplatelet therapy for 6 versus 18 months after biodegradable polymer drug-eluting stent implantation. Jacc-Cardiovasc Inte. (2017) 10(12):1189–98. 10.1016/j.jcin.2017.04.01928641838

[20] LongJ ZengF WangL YiC ChenQ ZhaoH. Gender-specific cardiovascular outcomes in patients undergoing percutaneous coronary intervention in Chinese populations. BMC Cardiovasc Disord. (2020) 20(1):280. 10.1186/s12872-020-01563-532517811 PMC7285452

[21] HessCN McCoyLA DuggiralaHJ TavrisDR O'CallaghanK DouglasPS Sex-based differences in outcomes after percutaneous coronary intervention for acute myocardial infarction: a report from TRANSLATE-ACS. J Am Heart Assoc. (2014) 3(1):e000523. 10.1161/JAHA.113.00052324510115 PMC3959683

[22] WolnyR MaeharaA LiuY ZhangZ MintzGS RedforsB The obesity paradox revisited: body mass index and -long-term outcomes after PCI from a large pooled patient-level database. Eurointervention. (2020) 15(13):1199–208. 10.4244/EIJ-D-19-0046731659983

[23] Feijóo-BandínS Aragón-HerreraA Moraña-FernándezS Anido-VarelaL TarazónE Roselló-LletíE Adipokines and inflammation: focus on cardiovascular diseases. Int J Mol Sci. (2020) 21(20):7711. 10.3390/ijms2120771133081064 PMC7589803

[24] FranekE PaisP BasileJ NicolayC RahaS HickeyA General versus central adiposity as risk factors for cardiovascular-related outcomes in a high-risk population with type 2 diabetes: a *post hoc* analysis of the REWIND trial. Cardiovasc Diabetol. (2023) 22(1):52. 10.1186/s12933-023-01757-z36899386 PMC9999507

[25] HayesSN TweetMS AdlamD KimESH GulatiR PriceJE Spontaneous coronary artery dissection: jACC state-of-the-art review. J Am Coll Cardiol. (2020) 76(8):961–84. 10.1016/j.jacc.2020.05.08432819471

