# Supplementary material for: Percutaneous coronary intervention in patients aged ≤40 years: a 10-year single-centre cohort study
Source: Front Cardiovasc Med. 2026 Mar 23;13:1748236. doi: 10.3389/fcvm.2026.1748236 (PMC13050886; doi:10.3389/fcvm.2026.1748236)
Supplement: Supplementary file 1 [file Datasheet1.docx]

**Supplementary Table 1. MACCE rates by SYNTAX score categories in patients ≤ 40 years**

| SYNTAX group | Patients (n) | 5-year MACCE (%) | Incidence rate (/1000 pt-yr) |
| --- | --- | --- | --- |
| 0–22 | 208 | 9.4 | 19.1 |
| 23–32 | 76 | 21.3 | 40.8 |
| ≥ 33 | 28 | 28.6 | 54.2 |

P for trend = 0.002 (Cox trend test). MACCE: major adverse cardiovascular and cerebrovascular events.

**Supplementary Table 2. AHA lesion-type distribution**

| **Type** | **n** | **%** |
| --- | --- | --- |
| A | 98 | 31 |
| B1 | 112 | 36 |
| B2 | 78 | 25 |
| C | 24 | 8 |

**Supplementary Table 3. Total MACCE burden (first and recurrent events) during 1,620 patient-years of follow-up**

| Endpoint | Count | Rate per 1,000 patient-years |
| --- | --- | --- |
| First MACCE | 45 | 27.8 |
| Additional recurrences | 36 | 22.2 |
| Total MACCE (first + recurrent) | 81 | 50.0 |

MACCE = major adverse cardiovascular and cerebrovascular events.

**Supplementary Table 4** Four-Step, Lifetime-Risk Care Pathway for Patients ≤ 40 Years Undergoing PCI

| **Step** | **Key Intervention** | **Target / Tool** |
| --- | --- | --- |
| 1 | Systematic risk-factor & family screening | Diabetes, smoking, premature CAD history |
| 2 | Imaging-guided PCI with DES | IVUS/OCT + radial-first approach |
| 3 | Intensive lipid control | LDL-C < 1.0 mmol/L; add PCSK9 inhibitor if needed |
| 4 | Prolonged antiplatelet therapy | DAPT ≥ 12 months + digital adherence support |
